# Surgical strategies for chondral defects of the patellofemoral joint: a systematic review

**DOI:** 10.1186/s13018-022-03419-4

**Published:** 2022-12-05

**Authors:** Filippo Migliorini, Alice Baroncini, Andreas Bell, Christian Weber, Frank Hildebrand, Nicola Maffulli

**Affiliations:** 1 Department of Orthopaedic, Trauma, and Reconstructive Surgery, RWTH University Hospital, Pauwelsstraße 30, 52074 Aachen, Germany; 2 Department of Orthopaedic and Trauma Surgery, Eifelklinik St. Brigida, 52152 Simmerath, Germany; 3 Department of Medicine, Surgery and Dentistry, University of Salerno, 84081 Baronissi, SA Italy; 4 School of Pharmacy and Bioengineering, Keele University Faculty of Medicine, Stoke on Trent, ST4 7QB England; 5 Barts and the London School of Medicine and Dentistry, Centre for Sports and Exercise Medicine, Queen Mary University of London, Mile End Hospital, London, E1 4DG England

**Keywords:** Knee, Patellofemoral, Chondral defects, AMIC, mACI

## Abstract

**Background:**

The management of chondral defects of the patellofemoral joint is debated, and definitive evidence is lacking. This study systematically updated and summarised the current literature on the surgical management of isolated chondral defects of the patellofemoral joint, discussing techniques, outcome, pitfalls, and new frontiers.

**Methods:**

This systematic review was conducted according to the 2020 PRISMA statement. In August 2022, PubMed, Web of Science, Google Scholar, and Embase databases were accessed with no time constrain. All the clinical studies investigating the surgical management of chondral defects of the patellofemoral joint were retrieved. Articles which reported data on patients with advanced to severe osteoarthritis were not eligible. Only studies with a minimum 24 months follow-up were considered. Studies which mixed results of patellofemoral and tibiofemoral joints were not considered.

**Results:**

Data from 10 studies (692 procedures) were retrieved. The mean follow-up was 46.9 ± 18.2 months. The mean age of the patients was 34.0 ± 6.1 years, and the mean BMI was 25.9 ± 0.8 kg/m^2^. The mean duration of symptoms before the index surgery was 81.0 ± 24.0 months. The mean defect size was 3.8 ± 0.8 cm^2^. All the PROMs improved from baseline to last follow-up: VAS 0–10 (*P* = 0.04), Tegner (*P* = 0.02), Lysholm (*P* = 0.03), and International Knee Documentation Committee (*P* = 0.03). The rate of hypertrophy was 5.6% (14 of 251), the rate of progression to total knee arthroplasty was 2.4% (2 of 83), the rate of revision was 16.9% (29 of 136), and the rate of failure was 13.0% (16 of 123).

**Conclusion:**

Current surgical strategies may be effective to improve symptoms deriving from chondral defects of the patellofemoral joint. The limited and heterogeneous data included for analysis impact negatively the results of the present study. Further clinical studies are strongly required to define surgical indications and outcomes, and the most suitable technique.

## Introduction

Chondral defects of the knee are common [1, 2]. Hyaline cartilage has limited regenerative capacities, and residual chondral defects are common [3, 4]. Non-symptomatic chondral defects are identified in up to 72% of patients undergo knee arthroscopy [5, 6]. Symptomatic chondral defects reduce considerably the quality of life and the participation to recreational activities of affected patients [7]. The management of patients with chondral defects is controversial, with unpredictable outcomes [8, 9]. Within the knee, chondral defects of the patellofemoral joint are common [10]. Chondral defects of the patellofemoral joint can lead to persistent anterior knee pain, and, if left untreated, to early osteoarthritis [7, 11, 12]. Bone marrow stimulating surgical strategies, such as microfractures, are commonly performed for smaller defects [8, 9]. Autologous Matrix-Induced Chondrogenesis (AMIC) represent a further evolution of microfractures: a resorbable membrane is placed over the defect to stabilise the bone marrow-derived blood clot [13]. Autologous chondrocyte implantation (ACI), first generation (periosteal patch, pACI), second generation (chondral patch, cACI), and third generation (matrix-induced ACI, mACI), has been widely performed in the patellofemoral joint [14–16]. Current evidence on the surgical management of chondral defects of the patellofemoral joint is limited and mainly arises from studies which combined results with those of the femorotibial joint [17–20]. Few clinical trials which exclusively focused on the management of isolated chondral defects of the patellofemoral joint have been published [21–30]. This study systematically updated and summarised the current evidence on the surgical management of isolated chondral defects of the patellofemoral joint, discussing techniques, patient reported outcome measures (PROMs), pitfalls, and new frontiers.

## Materials and methods

### Eligibility criteria

All the clinical trials investigating the surgical management of chondral defects of the patellofemoral joint were accessed. Given the authors’ language capabilities, articles in English, German, Italian, French, and Spanish were eligible. Only level I to IV of evidence, according to the Oxford Centre of Evidence-Based Medicine [31], were considered. Reviews, opinions, letters, and editorials were not considered. Only studies published in peer reviewed journals were considered. Animal, in vitro, biomechanics, computational, and cadaveric studies were not eligible. Articles which reported data on patients with advanced to severe osteoarthritis were not eligible. Studies which evaluated only the morphological quality of the newly formed cartilage were not eligible, nor were those reporting data from patients who previously underwent total knee arthroplasty (TKA). Only studies which reported the outcomes of patients undergoing surgical management for isolated chondral injuries of the patellofemoral joint (retropatellar, trochlear, or both) were considered. Only studies with a minimum 24 months follow-up were eligible. Studies which mixed results of patello- and tibiofemoral joints were not considered, nor were those which did not report quantitative data on the outcomes of interest.

### Search strategy

This systematic review was conducted according to the Preferred Reporting Items for Systematic Reviews and Meta-Analyses: the 2020 PRISMA statement [32]. The PICOT framework was followed for the initial search:

- P (Problem): chondral defects of the patella;
- I (Intervention): surgical intervention;
- C (Comparison): baseline to follow-up improvement;
- O (Outcomes): PROMs and complications;
- T (Timing): minimum 24 months follow-up.

In August 2022 PubMed, Web of Science, Google Scholar, and Embase databases were accessed with no time constrain. The following keywords were used in combination using the Boolean operators AND/OR: *knee, patella, patellofemoral, kneecap, chondral, cartilage defect, ailment, injury, damage, chondropathy, management, treatment, graft, surgery, intervention, patient reported outcome measures, PROMs, IKCD, Lysholm, Tegner, VAS, visual analogue scale, pain, function, joint, outcome, failure, reoperation, and revision*.

### Selection and data collection

The database search was conducted by two authors (F.M & A.B.) independently. All the resulting titles were carefully inspected and, if suitable, the abstract was accessed. The full-text of the abstracts which matched the topic was accessed. The bibliography of the full-text articles was also screened by hand to identify further articles. Disagreements were debated and the final decision was made by a third author (**).

### Data items

Two authors (F.M & A.B.) independently conducted data extraction. Generalities of the included studies were retrieved: author and year, journal, study design, mean length of the follow-up, mean age of the patients, mean length of the symptoms prior the index procedure, and mean defect size. Data with regard to the following PROMs were collected at baseline and at last follow-up: Visual Analogue Scale (VAS), Tegner Activity Scale [33], Lysholm Knee Scoring Scale [34], and International Knee Documentation Committee (IKDC) [35]. Data on the following complications were also collected: rates of hypertrophy, failure, and revision surgery. The rate of progression to TKA was also retrieved. Patients with persistent symptoms of chondral damage were considered as failures.

### Study risk of bias assessment

The study risk of bias assessment was performed by two authors (F.M & A.B.) independently. The risk of bias graft tool of the Review Manager software (The Nordic Cochrane Collaboration, Copenhagen) was used. The following biases were considered for analysis: selection, detection, reporting, attrition, and other source of bias.

### Synthesis methods

The statistical analyses were conducted by the main author (F.M.) using the IBM SPSS Software. For continuous data, the mean difference (MD) and standard error (SE) were evaluated. The confidence intervals (CI) were set at 95% in all the comparisons. The t test was also performed, with values of P < 0.05 considered statistically significant.

## Results

### Study selection

**Fig. 1:**
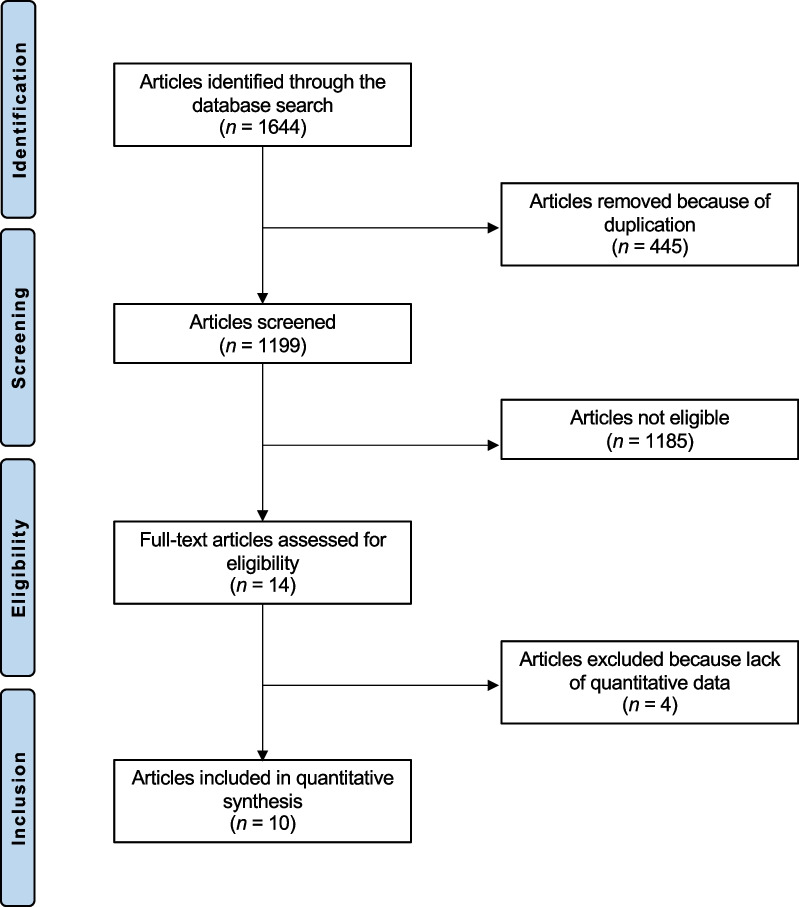
Flow chart of the literature search

A total of 1644 articles were identified during the preliminary database search. Of them, 445 were excluded as they were duplicates. A further 1185 studies were excluded with reason: not focusing on patellofemoral joint (*N* = 901), study type/design (*N* = 197), and mixed patellofemoral and tibiofemoral data (*N* = 41), including patients with osteoarthritis or only evaluated the quality of the new formed cartilage (*N* = 34), language limitations (*N* = 9), and short follow-up (*N* = 3). Finally, further 4 studies were excluded as they did not report quantitative data under the outcomes of interest. The literature search resulted in 10 articles (Fig. 1).

### Study risk of bias assessment

**Fig. 2:**
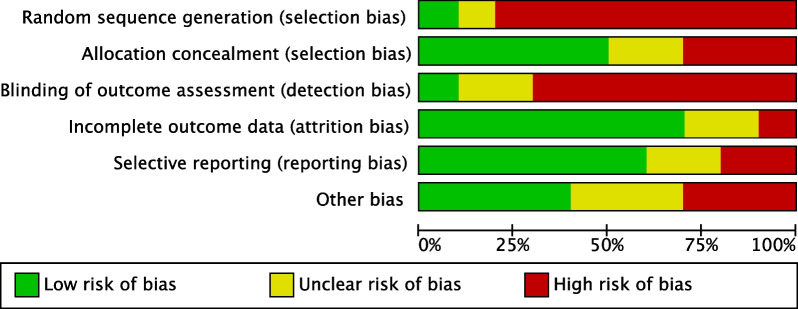
Methodological quality assessment

As a result of the lack of random sequence generation in most studies, the risk of bias tool evidenced a moderate risk of selection bias. Assessor blinding was seldom performed; thus, the risk of detection bias was moderate-high. However, the overall good quality of the investigations led to a moderate to low risk of attrition and reporting biases. The risk of other bias was moderate. Concluding, the risk of bias graph evidenced a moderate to low risk of publication bias (Fig. 2).

### Study characteristics and results of individual studies

Data from 692 procedures were retrieved. The mean follow-up was 46.9 ± 18.2 months. The mean age of the patients was 34.0 ± 6.1 years, and the mean BMI was 25.9 ± 0.8 kg/m^2^. The mean duration of the symptoms before the index surgery was 81.0 ± 24.0 months. The mean defect size was 3.8 ± 0.8 cm^2^. Generalities of the studies are shown in Table 1.

**Table 1:** Generalities and patient baseline of the included studies

Author, year	Journal	Design	Follow-up (months)	Type of treatment	Patients (n)	Mean age
Buda et al. [21]	*Eur J Orthop Surg Traumatol*	Retrospective	48.0	ACI & BMC	28	38.0
Ebert et al. [22]	*Am J Sports Med*	Prospective	24.0	ACI III generation	10	39.0
				ACI III generation	13	36.0
				ACI III generation	9	38.0
				ACI III generation	15	37.0
Macmull et al. [23]	*Int Orthop*	Prospective	45.0	ACI II generation	25	35.0
			35.3	ACI III generation	23	35.0
Meyerkort et al. [24]	*Knee Surg Sports Traumatol Arthrosc*	Prospective	60.0	ACI III generation	23	42.0
Migliorini et al. [25]	*LIFE*	Prospective	43.7	AMIC	52	29.5
			39.5	Microfractures	31	31.3
Niemeyer et al. [26]	*Am J Sports Med*	Retrospective	38.0	ACI I generation	52	34.0
				ACI III generation	315	
Teo et al. [27]	*Clin Orthop Relat Res*	Retrospective	24.0	ACI I generation	20	16.8
				ACI & BMC	3	
Tradati et al. [28]	*J Clin Med*	Retrospective	68.2	AMIC	14	38.4
Von Keudell et al. [29]	*Cartilage*	Prospective	88.0	ACI I generation	30	32.0
Waltenspül et al. [30]	*Cartilage*	Prospective	49.2	AMIC	29	27.9

*BMC* bone marrow concentrate, *ACI* autologous chondrocyte implantation, *AMIC* autologous matrix-induced chondrogenesis

### Results of syntheses

All the PROMs improved from baseline to last follow-up (Table 2): VAS 0–10 (− 1.8; *P* = 0.04), Tegner (+ 1.8; *P* = 0.02), Lysholm (+ 13.5; *P* = 0.03), and IKDC (+ 23.5; *P* = 0.03).

**Table 2:** Results of PROMs

Endpoint	Baseline	Last FU	MD	SE	95%CI	P
VAS	5.3 ± 1.3	3.5 ± 0.9	− 1.8	0.077	− 1.95 to − 1.64	0.04
Tegner	2.4 ± 0.2	4.2 ± 0.8	+ 1.8	0.045	1.71 to 1.88	0.02
Lysholm	55.0 ± 7.1	68.5 ± 5.9	+ 13.5	0.450	12.61 to 14.38	0.03
IKDC	50.1 ± 7.3	73.6 ± 12.2	+ 23.5	0.689	22.14 to 24.85	0.03

The rate of hypertrophy was 5.6% (14 of 251), the rate of progression to TKA was 2.4% (2 of 83), the rate of revision was 16.9% (29 of 136), and the rate of failure was 13.0% (16 of 123).

## Discussion

According to the main findings of the present study, current surgical strategies for chondral defects of the patellofemoral joint may be effective to improve symptoms. VAS, Tegner, Lysholm, and IKDC scores statistically significantly improved from baseline to last follow-up. The improvement of the PROMs overcome the minimum clinically important difference (MCID) [36–40]. However, the rate of complications was relatively high. The rate of hypertrophy was 5.6%, the rate of patients undergoing TKA was 2.4% (2 of 83), the rate of revision was 16.9% (29 of 136), and the rate of failure was 13.0% (16 of 123). Few authors reported the rate of complications: we assume that some authors did not report clearly whether complication were found. This may underestimate the number of patients who had experienced no complication.

Among the included studies, several techniques for cartilage regeneration have been used. PACI has been widely used to address chondral defects. Cultured autologous chondrocytes, harvested from healthy cartilage, are injected under a periosteal flap sutured over the defect [41]. PACI was burdened by a high rate of hypertrophy, which was attributed to the use of the periosteal flap [16, 42–46]. Indeed, in the present study, graft hypertrophy was almost seen only in patients who underwent pACI [26, 27]. To overcome this complication, collagen-membrane cover ACI (cACI) has been introduced, substituting the periosteal flap with a resorbable membrane [23, 47]. CACI evolved in mACI, in which the harvested and cultured autologous chondrocytes are seeded directly on a biodegradable scaffold, either a collagen type I/III matrix or hyaluronan matrix membrane [48]. The loaded matrix allows chondrocytes expansion and is secured with fibrin into the chondral defect in a second step surgery. Compared to the previous generation, mACI allows less invasive approaches (mini-arthrotomy or arthroscopy), avoids graft suture, and allows shorter surgical time [48]. Despite the good results in PROMs in long-term studies [49], these techniques are technically demanding, require two different surgical interventions, and are burdened by donor site morbidity, higher costs, and long recovery times [27, 48]. AMIC avoids cartilage harvest and external expansion, and is performed in a single surgical session. Different to ACI, which requires expanded autologous chondrocytes, AMIC exploits the potential of bone marrow-derived mesenchymal stem cells.

Most authors performed the procedures in patients with patellofemoral instability [21, 22, 26–30]. Up to 96% of patients following a patellar dislocation demonstrated cartilage damage at the patellofemoral joint [50–54]. Approximately 85% of patients following a patellar dislocation demonstrated focal patellar chondropathy at MRI, while 47% evidenced chondral damage in the trochlea [55–60]. The medial chondral facet and median crest of the patella were more commonly affected than the lateral patellar facet and femoral trochlea [51, 61–70]. Whether to combine a chondral procedure with a proximal or distal alignment to restore the appropriate patellar tracking is unclear. Patellofemoral instability is multifactorial, and its management is challenging [71]. The rate of re-dislocation after conservative management of first patellar dislocation ranges from 15 to 71% [72–83]. Surgery is reserved for patients who demonstrate loose bodies or large osteochondral defects [84–86]. There is a growing trend to manage surgically the first patellar dislocation [87, 88]. Previous investigations compared surgical versus conservative management for first patellofemoral dislocations, suggesting that patients may benefit from surgery immediately after the first acute patellar dislocation [89–98]. A recent meta-analysis found that the risk of re-dislocation was 2.44 times greater in the conservative group, with a 10% worse Kujala score at approximately 5 years follow-up compared to a group of patients who underwent immediate surgery [99]. However, the management of the first patellar dislocation remains unclear [100], and international recommendations are lacking.

This systematic review presents several limitations which impact the reliability of the conclusions. In the current literature, several studies reported data on chondral procedures in the patellofemoral joint; however, most authors did not focus exclusively on the patellofemoral joint. Evidence focusing exclusively on the patellofemoral joint is limited and heterogeneous. Studies which focused on chondral procedures for patellar defects used different surgical procedures, including AMIC [25, 28, 30], pACI [26, 27, 29], cACI [23], mACI [22–24, 26], and ACI augmented with bone marrow concentrate [21, 27]. In this respect, results from the present investigation are not fully generalisable. Additional between studies heterogeneities should be discussed. Most authors performed the chondral procedures in patients with patellofemoral instability. Patients who experienced acute or recurrent patellar dislocations have different patterns of chondral injuries [54, 101, 102]. Several risk factors which predispose to patellofemoral instability have been described, such as patella alta, malalignment syndromes, ligamentous hyperlaxity, and dysplasia [103–106]. Recent studies demonstrated that most patients with patellofemoral instability have two or more coexistent pathoanatomical risk factors which synergistically predispose to the dislocation. Therefore, the results are strongly influenced by the underlying subjective pathoanatomical susceptibility to instability. Future investigations should clarify the outcome of combined patellofemoral and chondral procedures, to establish the proper surgical management. Furthermore, some authors performed the chondral procedures in isolation [22, 25], while some others combined the procedure with proximal or distal alignments to restore physiological patellar tracking [21, 22, 26–29]. Eight studies focused on isolated retropatellar chondral injuries [22, 23, 25–30], one only on isolated trochlear injuries [22], and two reported data from mixed locations [21, 24]. Two studies enhanced ACI with autologous bone marrow aspirate concentrate [21, 27]. Moreover, most studies used a collagenic I/III porcine derived membrane, while Buda et al. 2018 [21] used a hyaluronic membrane. Most authors considered only patients with no previous chondral intervention on the knee [25, 29]; other authors considered also revision settings [22, 23, 26]. Finally, most authors included only patients with single unipolar patellofemoral joint focal defects [21, 22, 25, 28, 29], while others included also patients with mixed locations [23]. The impact of these variables on the surgical outcomes is unknown. However, given the lack of quantitative data available in the literature, further subgroup analyses were not possible. Given these limitations in original studies, additional subgroups analyses were not possible, and results from the present study must be interpreted with caution. Further clinical studies to define eligibility criteria, surgical indications and techniques are strongly required.

## Conclusions

Surgical strategies may be effective to improve symptoms of chondral defects of the patellofemoral joint. The rate of complications was relatively high. The limited and heterogeneous data included for analysis impact negatively the generalisability of the results of the present study. Further clinical studies are strongly required to define surgical indications and outcomes, and the most suitable technique.

## Data Availability

The datasets generated during and/or analysed during the current study are available throughout the manuscript.

## Abbreviations

MCID: Minimum clinically important difference
BMC: Bone marrow concentrate
ACI: Autologous chondrocyte implantation
AMIC: Autologous matrix-induced chondrogenesis
VAS: Visual analogue scale
IKDC: International Knee Documentation Committee
MD: Mean difference
SE: Standard error
CI: Confidence intervals
AMIC: Autologous Matrix-Induced Chondrogenesis
ACI: Autologous chondrocyte implantation
pACI: Periosteal patch autologous chondrocyte implantation
cACI: Chondral patch autologous chondrocyte implantation
mACI: Matrix-induced autologous chondrocyte implantation
PROMs: Patient reported outcome measures
TKA: Total knee arthroplasty

## References

[1] Richter DL, Schenck RC, Wascher DC (2016). Knee articular cartilage repair and restoration techniques: a review of the literature. Sports Health.

[2] Maheshwer B, Polce EM, Paul K (2021). Regenerative potential of mesenchymal stem cells for the treatment of knee osteoarthritis and chondral defects: a systematic review and meta-analysis. Arthroscopy.

[3] Atala A, Irvine DJ, Moses M (2010). Wound healing versus regeneration: role of the tissue environment in regenerative medicine. MRS Bull.

[4] Buckwalter JA (2002). Articular cartilage injuries. Clin Orthop Relat Res.

[5] Curl WW, Krome J, Gordon ES (1997). Cartilage injuries: a review of 31,516 knee arthroscopies. Arthroscopy.

[6] Figueroa D, Calvo R, Vaisman A (2007). Knee chondral lesions: incidence and correlation between arthroscopic and magnetic resonance findings. Arthroscopy.

[7] Heir S, Nerhus TK, Rotterud JH (2010). Focal cartilage defects in the knee impair quality of life as much as severe osteoarthritis: a comparison of knee injury and osteoarthritis outcome score in 4 patient categories scheduled for knee surgery. Am J Sports Med.

[8] Filardo G, Kon E, Berruto M (2012). Arthroscopic second generation autologous chondrocytes implantation associated with bone grafting for the treatment of knee osteochondritis dissecans: Results at 6 years. Knee.

[9] Bertho P, Pauvert A, Pouderoux T (2018). Treatment of large deep osteochondritis lesions of the knee by autologous matrix-induced chondrogenesis (AMIC): preliminary results in 13 patients. Orthop Traumatol Surg Res.

[10] Aroen A, Loken S, Heir S (2004). Articular cartilage lesions in 993 consecutive knee arthroscopies. Am J Sports Med.

[11] Samim M, Smitaman E, Lawrence D (2014). MRI of anterior knee pain. Skelet Radiol.

[12] Figueroa D, Calvo Rodriguez R, Donoso R (2020). High-grade patellar chondral defects: promising results from management with osteochondral autografts. Orthop J Sports Med.

[13] de Girolamo L, Schonhuber H, Vigano M (2019). Autologous matrix-induced chondrogenesis (AMIC) and AMIC enhanced by autologous concentrated bone marrow aspirate (BMAC) allow for stable clinical and functional improvements at up to 9 years follow-up: results from a randomized controlled study. J Clin Med.

[14] Basad E, Wissing FR, Fehrenbach P (2015). Matrix-induced autologous chondrocyte implantation (MACI) in the knee: clinical outcomes and challenges. Knee Surg Sports Traumatol Arthrosc.

[15] Basad E, Ishaque B, Bachmann G (2010). Matrix-induced autologous chondrocyte implantation versus microfracture in the treatment of cartilage defects of the knee: a 2-year randomised study. Knee Surg Sports Traumatol Arthrosc.

[16] Bartlett W, Skinner JA, Gooding CR (2005). Autologous chondrocyte implantation versus matrix-induced autologous chondrocyte implantation for osteochondral defects of the knee: a prospective, randomised study. J Bone Joint Surg Br.

[17] Volz M, Schaumburger J, Frick H (2017). A randomized controlled trial demonstrating sustained benefit of Autologous Matrix-Induced Chondrogenesis over microfracture at five years. Int Orthop.

[18] Anders S, Volz M, Frick H (2013). A randomized, controlled trial comparing autologous matrix-induced chondrogenesis (AMIC(R)) to microfracture: analysis of 1- and 2-year follow-up data of 2 centers. Open Orthop J.

[19] Chung JY, Lee DH, Kim TH (2014). Cartilage extra-cellular matrix biomembrane for the enhancement of microfractured defects. Knee Surg Sports Traumatol Arthrosc.

[20] Sadlik B, Puszkarz M, Kosmalska L (2017). All-arthroscopic autologous matrix-induced chondrogenesis-aided repair of a patellar cartilage defect using dry arthroscopy and a retraction system. J Knee Surg.

[21] Buda R, Baldassarri M, Perazzo L (2019). A useful combination for the treatment of patellofemoral chondral lesions: realignment procedure plus mesenchymal stem cell-retrospective analysis and clinical results at 48 months of follow-up. Eur J Orthop Surg Traumatol.

[22] Ebert JR, Fallon M, Smith A (2015). Prospective clinical and radiologic evaluation of patellofemoral matrix-induced autologous chondrocyte implantation. Am J Sports Med.

[23] Macmull S, Jaiswal PK, Bentley G (2012). The role of autologous chondrocyte implantation in the treatment of symptomatic chondromalacia patellae. Int Orthop.

[24] Meyerkort D, Ebert JR, Ackland TR (2014). Matrix-induced autologous chondrocyte implantation (MACI) for chondral defects in the patellofemoral joint. Knee Surg Sports Traumatol Arthrosc.

[25] Migliorini F, Eschweiler J, Maffulli N (2021). Management of patellar chondral defects with autologous matrix induced chondrogenesis (AMIC) compared to microfractures: a four years follow-up clinical trial. Life (Basel).

[26] Niemeyer P, Pestka JM, Kreuz PC (2008). Characteristic complications after autologous chondrocyte implantation for cartilage defects of the knee joint. Am J Sports Med.

[27] Teo BJ, Buhary K, Tai BC (2013). Cell-based therapy improves function in adolescents and young adults with patellar osteochondritis dissecans. Clin Orthop Relat Res.

[28] Tradati D, De Luca P, Maione A (2020). AMIC-autologous matrix-induced chondrogenesis technique in patellar cartilage defects treatment: a retrospective study with a mid-term follow-up. J Clin Med.

[29] von Keudell A, Han R, Bryant T (2017). Autologous chondrocyte implantation to isolated patella cartilage defects. Cartilage.

[30] Waltenspul M, Suter C, Ackermann J, et al. 2021. Autologous matrix-induced chondrogenesis (AMIC) for isolated retropatellar cartilage lesions: outcome after a follow-up of minimum 2 years. Cartilage:19476035211021908. 10.1177/19476035211021908 PMC8808854 34116609

[31] Howick JCI, Glasziou P, Greenhalgh T, Carl Heneghan, Liberati A, Moschetti I, Phillips B, Thornton H, Goddard O, Hodgkinson M. 2011. The 2011 Oxford CEBM levels of evidence. Oxford Centre for Evidence-Based Medicine Available at https://www.cebmnet/indexaspx?o=5653.

[32] Page MJ, McKenzie JE, Bossuyt PM (2021). The PRISMA 2020 statement: an updated guideline for reporting systematic reviews. BMJ.

[33] Briggs KK, Lysholm J, Tegner Y (2009). The reliability, validity, and responsiveness of the Lysholm score and Tegner activity scale for anterior cruciate ligament injuries of the knee: 25 years later. Am J Sports Med.

[34] Lysholm J, Gillquist J (1982). Evaluation of knee ligament surgery results with special emphasis on use of a scoring scale. Am J Sports Med.

[35] Higgins LD, Taylor MK, Park D (2007). Reliability and validity of the International Knee Documentation Committee (IKDC) Subjective Knee Form. Joint Bone Spine.

[36] Agarwalla A, Liu JN, Garcia GH, et al. 2020. Return to sport following isolated lateral opening wedge distal femoral osteotomy. Cartilage:1947603520924775. 10.1177/1947603520924775 PMC8808905 32449382

[37] Jones KJ, Kelley BV, Arshi A (2019). Comparative effectiveness of cartilage repair with respect to the minimal clinically important difference. Am J Sports Med.

[38] Mostafaee N, Negahban H, Shaterzadeh Yazdi MJ (2020). Responsiveness of a Persian version of Knee Injury and Osteoarthritis Outcome Score and Tegner activity scale in athletes with anterior cruciate ligament reconstruction following physiotherapy treatment. Physiother Theory Pract.

[39] Todd KH, Funk KG, Funk JP (1996). Clinical significance of reported changes in pain severity. Ann Emerg Med.

[40] Kelly AM (1998). Does the clinically significant difference in visual analog scale pain scores vary with gender, age, or cause of pain?. Acad Emerg Med.

[41] Brittberg M, Lindahl A, Nilsson A (1994). Treatment of deep cartilage defects in the knee with autologous chondrocyte transplantation. N Engl J Med.

[42] Driesang IM, Hunziker EB (2000). Delamination rates of tissue flaps used in articular cartilage repair. J Orthop Res.

[43] Henderson I, Tuy B, Oakes B (2004). Reoperation after autologous chondrocyte implantation. Indications and findings. J Bone Joint Surg Br.

[44] Kreuz PC, Steinwachs M, Erggelet C (2007). Classification of graft hypertrophy after autologous chondrocyte implantation of full-thickness chondral defects in the knee. Osteoarthr Cartil.

[45] Micheli LJ, Browne JE, Erggelet C (2001). Autologous chondrocyte implantation of the knee: multicenter experience and minimum 3-year follow-up. Clin J Sport Med.

[46] Zeifang F, Oberle D, Nierhoff C (2010). Autologous chondrocyte implantation using the original periosteum-cover technique versus matrix-associated autologous chondrocyte implantation: a randomized clinical trial. Am J Sports Med.

[47] Niemeyer P, Salzmann G, Feucht M (2014). First-generation versus second-generation autologous chondrocyte implantation for treatment of cartilage defects of the knee: a matched-pair analysis on long-term clinical outcome. Int Orthop.

[48] Brittberg M (2010). Cell carriers as the next generation of cell therapy for cartilage repair: a review of the matrix-induced autologous chondrocyte implantation procedure. Am J Sports Med.

[49] Makris EA, Gomoll AH, Malizos KN (2015). Repair and tissue engineering techniques for articular cartilage. Nat Rev Rheumatol.

[50] Guerrero P, Li X, Patel K (2009). Medial patellofemoral ligament injury patterns and associated pathology in lateral patella dislocation: an MRI study. Sports Med Arthrosc Rehabil Ther Technol.

[51] Nomura E, Inoue M, Kurimura M (2003). Chondral and osteochondral injuries associated with acute patellar dislocation. Arthroscopy.

[52] Sanders TG, Paruchuri NB, Zlatkin MB (2006). MRI of osteochondral defects of the lateral femoral condyle: incidence and pattern of injury after transient lateral dislocation of the patella. AJR Am J Roentgenol.

[53] Potter HG, Linklater JM, Allen AA (1998). Magnetic resonance imaging of articular cartilage in the knee. An evaluation with use of fast-spin-echo imaging. J Bone Joint Surg Am.

[54] Migliorini F, Pilone M, Eschweiler J (2022). High rates of damage to the medial patellofemoral ligament, lateral trochlea, and patellar crest after acute patellar dislocation: magnetic resonance imaging analysis. Arthroscopy.

[55] Zaidi A, Babyn P, Astori I (2006). MRI of traumatic patellar dislocation in children. Pediatr Radiol.

[56] Vollnberg B, Koehlitz T, Jung T (2012). Prevalence of cartilage lesions and early osteoarthritis in patients with patellar dislocation. Eur Radiol.

[57] Paakkala A, Sillanpaa P, Huhtala H (2010). Bone bruise in acute traumatic patellar dislocation: volumetric magnetic resonance imaging analysis with follow-up mean of 12 months. Skelet Radiol.

[58] Seeley M, Bowman KF, Walsh C (2012). Magnetic resonance imaging of acute patellar dislocation in children: patterns of injury and risk factors for recurrence. J Pediatr Orthop.

[59] Zhang GY, Zheng L, Feng Y (2015). Injury patterns of medial patellofemoral ligament and correlation analysis with articular cartilage lesions of the lateral femoral condyle after acute lateral patellar dislocation in adults: an MRI evaluation. Injury.

[60] Zheng L, Shi H, Feng Y (2015). Injury patterns of medial patellofemoral ligament and correlation analysis with articular cartilage lesions of the lateral femoral condyle after acute lateral patellar dislocation in children and adolescents: an MRI evaluation. Injury.

[61] Sallay PI, Poggi J, Speer KP (1996). Acute dislocation of the patella. A correlative pathoanatomic study. Am J Sports Med.

[62] Nomura E, Inoue M (2005). Second-look arthroscopy of cartilage changes of the patellofemoral joint, especially the patella, following acute and recurrent patellar dislocation. Osteoarthr Cartil.

[63] Saragaglia D, Banihachemi JJ, Refaie R (2020). Acute instability of the patella: is magnetic resonance imaging mandatory?. Int Orthop.

[64] Callewier A, Monsaert A, Lamraski G (2009). Lateral femoral condyle osteochondral fracture combined to patellar dislocation: a case report. Orthop Traumatol Surg Res.

[65] Maletius W, Lundberg M (1994). Refixation of large chondral fragments on the weight-bearing area of the knee joint: a report of two cases. Arthroscopy.

[66] Mashoof AA, Scholl MD, Lahav A (2005). Osteochondral injury to the mid-lateral weight-bearing portion of the lateral femoral condyle associated with patella dislocation. Arthroscopy.

[67] Megremis P, Megremis O, Margariti R (2019). Late repair, one year after a knee twisting injury, of a missed femoral trochlea osteochondral fragment, with bioabsorbable nails, in a 14-year-old boy. J Am Acad Orthop Surg Glob Res Rev.

[68] Stanitski CL (1995). Articular hypermobility and chondral injury in patients with acute patellar dislocation. Am J Sports Med.

[69] Stanitski CL, Paletta GA (1998). Articular cartilage injury with acute patellar dislocation in adolescents. Arthroscopic and radiographic correlation. Am J Sports Med.

[70] von Engelhardt LV, Raddatz M, Bouillon B (2010). How reliable is MRI in diagnosing cartilaginous lesions in patients with first and recurrent lateral patellar dislocations?. BMC Musculoskelet Disord.

[71] Fithian DC, Paxton EW, Stone ML (2004). Epidemiology and natural history of acute patellar dislocation. Am J Sports Med.

[72] Maenpaa H, Lehto MU (1997). Patellar dislocation. The long-term results of nonoperative management in 100 patients. Am J Sports Med.

[73] Moiz M, Smith N, Smith TO (2018). Clinical outcomes after the nonoperative management of lateral patellar dislocations: a systematic review. Orthop J Sports Med.

[74] Larsen E, Lauridsen F (1982). Conservative treatment of patellar dislocations. Influence of evident factors on the tendency to redislocation and the therapeutic result. Clin Orthop Relat Res.

[75] Arendt EA, Askenberger M, Agel J (2018). Risk of redislocation after primary patellar dislocation: a clinical prediction model based on magnetic resonance imaging variables. Am J Sports Med.

[76] Balcarek P, Oberthur S, Hopfensitz S (2014). Which patellae are likely to redislocate?. Knee Surg Sports Traumatol Arthrosc.

[77] Jaquith BP, Parikh SN (2017). Predictors of recurrent patellar instability in children and adolescents after first-time dislocation. J Pediatr Orthop.

[78] Zhang GY, Ding HY, Li EM (2019). Incidence of second-time lateral patellar dislocation is associated with anatomic factors, age and injury patterns of medial patellofemoral ligament in first-time lateral patellar dislocation: a prospective magnetic resonance imaging study with 5-year follow-up. Knee Surg Sports Traumatol Arthrosc.

[79] Hevesi M, Heidenreich MJ, Camp CL (2019). The recurrent instability of the patella score: a statistically based model for prediction of long-term recurrence risk after first-time dislocation. Arthroscopy.

[80] Christensen TC, Sanders TL, Pareek A (2017). Risk factors and time to recurrent ipsilateral and contralateral patellar dislocations. Am J Sports Med.

[81] Sillanpaa PJ, Peltola E, Mattila VM (2009). Femoral avulsion of the medial patellofemoral ligament after primary traumatic patellar dislocation predicts subsequent instability in men: a mean 7-year nonoperative follow-up study. Am J Sports Med.

[82] Yeoh CS, Lam KY (2016). Tibial tubercle to trochlear groove distance and index in children with one-time versus recurrent patellar dislocation: a magnetic resonance imaging study. J Orthop Surg (Hong Kong).

[83] Sanders TL, Pareek A, Hewett TE (2018). High rate of recurrent patellar dislocation in skeletally immature patients: a long-term population-based study. Knee Surg Sports Traumatol Arthrosc.

[84] Stefancin JJ, Parker RD (2007). First-time traumatic patellar dislocation: a systematic review. Clin Orthop Relat Res.

[85] Buchner M, Baudendistel B, Sabo D (2005). Acute traumatic primary patellar dislocation: long-term results comparing conservative and surgical treatment. Clin J Sport Med.

[86] Clark D, Metcalfe A, Wogan C (2017). Adolescent patellar instability: current concepts review. Bone Joint J.

[87] Fukushima K, Horaguchi T, Okano T (2004). Patellar dislocation: arthroscopic patellar stabilization with anchor sutures. Arthroscopy.

[88] Hing CB, Smith TO, Donell S (2011). Surgical versus non-surgical interventions for treating patellar dislocation. Cochrane Database Syst Rev.

[89] Apostolovic M, Vukomanovic B, Slavkovic N (2011). Acute patellar dislocation in adolescents: operative versus nonoperative treatment. Int Orthop.

[90] Bitar AC, Demange MK, D'Elia CO (2012). Traumatic patellar dislocation: nonoperative treatment compared with MPFL reconstruction using patellar tendon. Am J Sports Med.

[91] Camanho GL, Viegas Ade C, Bitar AC (2009). Conservative versus surgical treatment for repair of the medial patellofemoral ligament in acute dislocations of the patella. Arthroscopy.

[92] Christiansen SE, Jakobsen BW, Lund B (2008). Isolated repair of the medial patellofemoral ligament in primary dislocation of the patella: a prospective randomized study. Arthroscopy.

[93] Ji G, Wang S, Wang X (2017). Surgical versus nonsurgical treatments of acute primary patellar dislocation with special emphasis on the MPFL injury patterns. J Knee Surg.

[94] Lee HL, Yau WP (2017). Management of traumatic patellar dislocation in a regional hospital in Hong Kong. Hong Kong Med J.

[95] Petri M, Liodakis E, Hofmeister M (2013). Operative vs conservative treatment of traumatic patellar dislocation: results of a prospective randomized controlled clinical trial. Arch Orthop Trauma Surg.

[96] Regalado G, Lintula H, Kokki H (2016). Six-year outcome after non-surgical versus surgical treatment of acute primary patellar dislocation in adolescents: a prospective randomized trial. Knee Surg Sports Traumatol Arthrosc.

[97] Sillanpaa PJ, Mattila VM, Maenpaa H (2009). Treatment with and without initial stabilizing surgery for primary traumatic patellar dislocation. A prospective randomized study. J Bone Joint Surg Am.

[98] Sillanpaa PJ, Maenpaa HM, Mattila VM (2008). Arthroscopic surgery for primary traumatic patellar dislocation: a prospective, nonrandomized study comparing patients treated with and without acute arthroscopic stabilization with a median 7-year follow-up. Am J Sports Med.

[99] Migliorini F, Driessen A, Quack V (2020). Surgical versus conservative treatment for first patellofemoral dislocations: a meta-analysis of clinical trials. Eur J Orthop Surg Traumatol.

[100] Vetrano M, Oliva F, Bisicchia S (2017). I.S.Mu.L.T. first-time patellar dislocation guidelines. Muscles Ligaments Tendons J.

[101] Migliorini F, Marsilio E, Oliva F (2022). Chondral injuries in patients with recurrent patellar dislocation: a systematic review. J Orthop Surg Res.

[102] Migliorini F, Marsilio E, Cuozzo F (2021). Chondral and soft tissue injuries associated to acute patellar dislocation: a systematic review. Life (Basel).

[103] Malagelada F, Rahbek O, Sahirad C (2018). Results of operative 4-in-1 patella realignment in children with recurrent patella instability. J Orthop.

[104] Katagiri H, Miyatake K, Watanabe T (2020). Validity of intraoperative observation of graft length change pattern for medial patellofemoral ligament reconstruction. J Orthop.

[105] Hadley CJ, Tucker BS, Lombardi NJ (2021). Combined MPFL reconstruction and tibial tubercle osteotomy for patellar instability: a retrospective review of 23 patients. J Orthop.

[106] Surendran S (2014). Patellar instability: changing beliefs and current trends. J Orthop.

